# The Effects of Cow-Milk Protein Supplementation in Elderly Population: Systematic Review and Narrative Synthesis

**DOI:** 10.3390/nu12092548

**Published:** 2020-08-23

**Authors:** Barbara Zanini, Anna Simonetto, Matilde Zubani, Maurizio Castellano, Gianni Gilioli

**Affiliations:** 1 Department of Clinical and Experimental Sciences, University of Brescia, Viale Europa, 11, I-25123 Brescia, Italy; maurizio.castellano@unibs.it; 2 AgroFood Lab, Department of Molecular and Translational Medicine, University of Brescia, Viale Europa, 11, I-25123 Brescia, Italy; anna.simonetto@unibs.it (A.S.); matilde.zubani@unibs.it (M.Z.); gianni.gilioli@unibs.it (G.G.)

**Keywords:** whey protein, casein, older people, muscle mass, sarcopenia, nutrition

## Abstract

Background. To review currently available evidence on the effect of cow-milk proteins supplementation (CPS) on health in the elderly. Methods. Five electronic databases (Pubmed, Web of Science, Embase, Cochrane Library, ClinicalTrials.gov) were searched for studies about CPS among older people. All types of publications were included, with the exception of systematic reviews, meta-analyses, opinion letters, editorials, case reports, conference abstracts and comments. An additional search in Google Scholar and a manual review of the reference lists were performed. Results. Overall, 103 studies were included. Several studies explored the role of CPS in the preservation or improvement of muscle mass among healthy subjects (40 studies) and pre-frail, frail or sarcopenic patients (14), with evidence of beneficial effects. Other studies assessed the effect of CPS on bones (12), cardiovascular disease (8), inflamm-aging (7), chronic pulmonary disease (4), neurocognitive function (4), and vaccines (2), with weak evidence of positive effects. Seven studies in the field of protein metabolism investigated the role of CPS as an important contributor to nutritional needs. Other investigational areas are considered in the last five studies. Conclusions. The beneficial effects of CPS in achieving aged-related nutritional goals, in preserving muscle mass and in recovering after hospitalization may be particularly relevant in the elderly.

## 1. Introduction

The World Health Organization declared the years between 2020 and 2030 as the Decade of Healthy Ageing. By the end of 2020, the number of people aged over 60 years old will surpass the number of children under 5 years old. Elderly people will globally increase from 1 billion in 2019 to 1.4 billion in 2030 (about 34% increase rate). By 2050, the proportion of people aged 60 years among the population is expected to be one in five [1]. Considering this demographic transition, the preservation of wellbeing is a crucial issue of ‘adding years to life’ [2].

Since the beginning of 2000, ‘healthy ageing’ has been defined as “a lifelong process optimising opportunities for improving and preserving health and physical, social and mental wellness, independence, quality of life and enhancing successful life-course transitions” [3].

An emerging condition affecting healthy aging is sarcopenia. In 2009, the International Sarcopenia Consensus Conference Working Group defined sarcopenia as “an age-related loss of skeletal muscle mass, with or without an increase in fat mass” [4]. More recently, sarcopenia has been defined by a European Consensus as “a muscle disease (muscle failure) rooted in adverse muscle changes that accrue across a lifetime” [5]. According to this new consensus, the key characteristics of sarcopenia are low muscle strength and reduced physical performance. Optimal care is crucial in the prevention and treatment of sarcopenia, because this condition is related to increased risk of fractures, impaired ability to perform activities of daily living, cardiovascular and respiratory diseases, cognitive impairment, loss of independence, and eventually death.

Scientific evidence suggests a central role of protein intake in preserving lean mass and preventing sarcopenia, but the definition of the best quantity and quality of protein sources is still an open issue [6,7,8].

Ageing is a plastic process, and it may affect nutritional requirements [9,10]. For example, basic science studies demonstrated that protein metabolism in the elderly is characterized by a high splanchnic extraction and a declining anabolic response to ingested proteins [11]. Lifestyle factors, such as high-quality diet, physical activity, little or no alcohol consumption and smoking avoidance, can influence the quality of ageing, improving wellbeing throughout the life span [9]. Taking into account these findings, the European Union Geriatric Medicine Society (EUGMS), in cooperation with other scientific organizations, appointed an international study group to review dietary protein needs with aging (PROT-AGE Study Group) [11]. According to PROT-AGE position paper, recommendations for dietary protein intake in healthy older adults are as follows:

- average protein intake for older people should range from 1.0 to 1.2 g/kg of body weight per day (while in young adults, the recommended intake is about 0.7–0.8 g/kg/day) [12];
- it must be taken into account that the feeding-associated anabolic threshold for dietary protein is higher in the elderly than in younger subjects, with the amount of protein required to reach it from a variety of foods being in the order of 25–30 g of protein per meal;
- dietary recommendations for protein intake in the elderly should consider, beyond quantity, also quality, protein source and timing of intake;
- best protein sources are rich in leucine;
- oral supplementation should be considered when dietary protein intake does not reach recommended goals [11].

Sources of animal proteins, such as meat, fish and poultry, are excellent for their essential amino-acid content, but their consumption may be impaired in the elderly, because of poor dentition, reduced appetite, or even anorexia, solid dysphagia, taste alteration, cost, and, when mobility is reduced, barriers in shopping and cooking [13,14]. Legumes and pulses are even good protein sources, but may enhance gastrointestinal functional disorders, such as slow gastric emptying, bloating, abdominal distention and diarrhea [15]. Dairy foods are rich in leucine, and they are available in many different forms, even soft and enriched with probiotics, but the weekly amount is usually restricted to 2–3 servings (excluding milk and yogurt), due to their fat content [16].

There is a large variety of protein oral supplements, mainly based on soy or cow-milk sources [17]. Among the latter, whey proteins (WP), a by-product of cheese making, should be regarded as one of the best sources for oral protein supplementation, for their high leucine content, fast digestibility and amino-acid availability (demonstrated in both young and old subjects) [18,19].

With regard to these considerations, we performed a systematic literature review, to investigate the role of cow-milk proteins (hence forward generally called “milk proteins”) supplementation in the elderly, exploring its effects on several health outcomes particularly relevant to older people (e.g., muscle and bone mass preservation, cognitive performance, cardiovascular risk factors). Our aim was to map the scientific evidence currently available, in order to evaluate which outcomes have already been widely targeted, and to identify those which still have to be studied in depth with new trials. We also aimed to assess the presence of knowledge gaps yet to be addressed. We expected to include studies with a high level of heterogeneity, so we considered it to be more appropriate to present the results of the systematic literature review as a narrative synthesis.

## 2. Materials and Methods

The systematic literature review started in April 2019, and it has been performed according to the Preferred Reporting Items for Systematic Review and Meta-Analyses (PRISMA) guidelines [20]. The review has been registered on PROSPERO—international prospective register of systematic reviews (CRD 42020137114).

### 2.1. Literature Search Strategy

Five electronic databases were systematically searched: PubMed, Web of Science, Embase, Cochrane Library, ClinicalTrials.gov. The strings used for the search in these databases were based on Medical Subject Heading (MeSH) terms, text keywords and Boolean operators, as follows:

PubMed: “Milk Proteins/administration and dosage” [MeSH] OR “Milk Proteins/adverse effects” [MeSH] OR “Milk Proteins/metabolism” [MeSH] OR “Milk Proteins/organization and administration” [MeSH] OR “Milk Proteins/pharmacokinetics” [MeSH] OR “Milk Proteins/pharmacology” [MeSH] OR “Milk Proteins/supply and distribution” [MeSH] OR “Milk Proteins/therapeutic use” [MeSH] OR “Milk Proteins/therapy” [MeSH] OR “Milk Proteins/toxicity” [MeSH]) AND “aged” [MeSH].

*Web of Science*: (“milk proteins” OR “whey proteins” OR “caseins”) AND (“older” OR “elderly”) in the categories Nutrition Dietetics, Endocrinology Metabolism, Medicine Research Experimental, Geriatrics Gerontology, Toxicology, Chemistry Medicinal, Gastroenterology Hepatology, Medicine General Internal, Pathology, Integrative Complementary Medicine, Gerontology, Rehabilitation.

*Embase*: (“milk proteins” OR “whey proteins” OR “caseins”) AND (“aged” OR “old” OR “elderly”) in titles, abstracts or keywords.

*Cochrane Library*: (“milk proteins” OR “whey proteins” OR “caseins”) AND (“aged” OR “old” OR “elderly”) in title, abstract or keywords.

*ClinicalTrial.gov* was checked for ongoing or unpublished trials: “ageing” as condition or disease, “milk proteins” as other terms.

A search in Google Scholar was also performed using the terms “milk proteins” AND “elderly”.

The whole literature search was limited to interventions on human beings, and the language restricted to English, whereas no restriction on publication year was applied.

The reference lists of the eligible studies were manually searched for additional articles.

### 2.2. Study Selection

Two investigators (B.Z. and M.Z.) independently selected the studies to be included in the review, on the basis of pre-defined eligibility criteria and screened titles and abstracts. One author (BZ) checked full texts for final inclusion. Any disagreement was resolved by discussion and re-examination of the studies by two investigators (B.Z. and M.Z.).

To be included in the review, studies had to consider: (i) subjects aged 60 years or more, or (ii) menopausal/postmenopausal women. We excluded studies whose target groups were children, adolescent, young or adults, or if an aged-based analysis in subjects over 60 years was not provided. In all included studies, at least one intervention arm had to include supplementation with cow-milk proteins, caseins, whey proteins or bioactive cow-milk peptides. We included randomized clinical trials (RCTs), observational, cross-sectional, cohort and case-control studies. We excluded opinion letters, editorials, case reports, conference abstracts, and comments. We also excluded systematic reviews and meta-analyses, after having manually searched their references lists to be sure that all relevant studies were already included in our review.

Studies are presented as a narrative synthesis, organized in sub-sections according to the different health outcomes. We reported the main features of each study, including information about participants, intervention and principal endpoints.

## 3. Results

**Figure 1:**
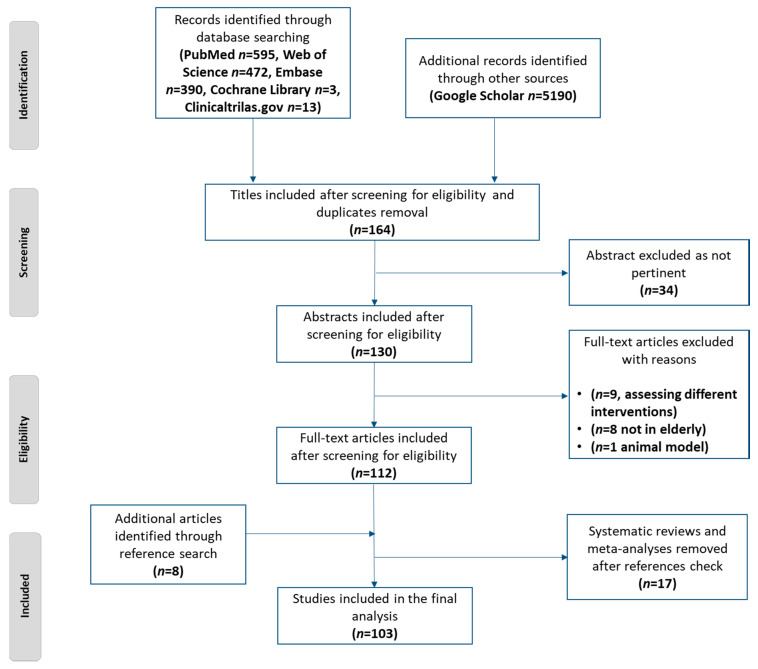
Preferred Reporting Items for Systematic Review and Meta-Analyses (PRISMA) flow diagram of the literature screening process.

**Figure 2:**
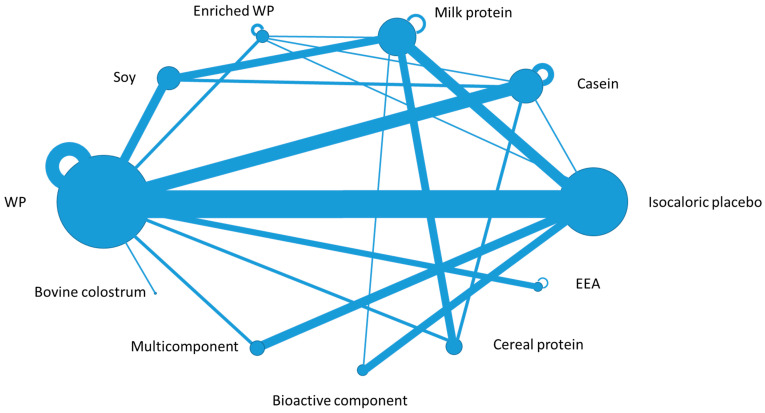
The network plot of the 11 intervention arms included in the randomized clinical trials (82 studies). The intervention arms are coded in the plot as nodes: each node size is proportional to the number of direct comparisons involving each intervention. The 20 lines between nodes represent direct comparisons driven by the trials; the line thickness is proportional to the number of studies where the direct comparison was performed. The additional semicircles over five nodes represent comparisons of different dosages within the same intervention (WP: Whey Proteins, EEA: Essential Amino Acids).

According to the predefined search strategy, 1473 studies were obtained from the 5 databases and 5190 studies from Google Scholar. The flow diagram of the screening process is reported in Figure 1. At the end of the screening process, 103 studies were included in the narrative synthesis, and out of them, 82 were RCTs. Figure 2 provides the network plot of the 11 intervention arms considered in the RCTs included in the review.

### 3.1. Muscle Related Endpoints among Healthy Subjects (40 Studies)

In this section we considered only studies recruiting old healthy subjects, aged 60 years or more, not specifically addressed as ‘patients’. Subjects recruited in the studies of this section were not sarcopenic, hospitalized, frail or at risk for frailty). Ageing is commonly associated with a loss in skeletal muscle mass, and epidemiological research has assessed a strong association between muscle strength and risk for developing age-related diseases and mortality [21]. Protein intake with diet is crucial in stimulating muscle protein synthesis (MPS or anabolism), and in decreasing muscle protein breakdown (catabolism). Proteins in diet can affect the extent and the duration of muscle anabolism, both in young and elderly people [22].

In both elderly women and men, the leucine content of dietary protein seems to be the primary determinant of postprandial MPS [23,24]. Leucine is the most potent amino acid, because of its role as a signal in activation of protein anabolism. Leucine is a strong activator of protein synthesis, but its anabolic threshold is impaired in the elderly—a phenomenon called ‘anabolic resistance’ [25]. As a result, during ageing, a higher content of leucine intake may be necessary to reach an effective anabolic leucinemia level, correctly stimulating muscle anabolism [26,27]. Interestingly, leucinemia-mediated post prandial MPS is not affected by the previous habituation to a low or high protein content of diet in the elderly (0.7 g/kg vs. 1.5 g/kg) [28]. Moreover, a study among 12 healthy older and 12 young men confirmed a good MPS response in both groups, following the ingestion of a meal-like amount of dietary proteins (20 g casein) and carbohydrates (40 g) [29].

In Table 1, we gathered fifteen studies aiming to identify the best protein supplementation able to optimize digestion, absorption, post-prandial peak in leucinemia level, body protein balance, muscle protein synthesis and accretion [30,31,32,33,34,35,36,37,38,39,40,41,42,43,44]. These studies mainly assessed the effect of a single dose administration of different protein supplementations. Despite several differences in intervention arms, these studies provide evidence of the superiority of WP, in comparison to casein, in stimulating MPS, with better results with leucine-enriched WP supplements and higher WP doses (35 g).

In recent years, several RCTs investigated the effects of different multicomponent supplementation, consisting of WP, in association with other proteins, macro- or micro-nutrients:

- co-ingestion of carbohydrates and fats with 21 g of leucine enriched WP did not affect the improvement of MPS rates, among 45 nonsarcopenic older men [45];
- a high WP-leucine- and vitamin D-enriched supplement (21 g protein in 150 Kcal/serving, 10 servings per week) was effective in preserving muscle mass during intentional weight reduction in association with regular physical activity, among 80 obese older adults [46];
- leucine-enriched WP (21 g) and vitamin D (800 IU) daily supplementation before breakfast enhanced post prandial MPS (acute effect) and muscle mass (long term effect) in 24 healthy elderly men in a ‘proof of principle’ trial [47];
- a multi-ingredient supplementation consisting of 30 g WP, 2.5 g creatine, 500 IU vitamin D, 400 mg calcium and 1500 mg n-3 PUFA was tested with and without exercise versus placebo and was effective in increasing both muscle strength and mass among 49 older men [48];
- co-ingestion of milk fat (26.7 g) did not affect the raise in plasma amino-acids and MPS after the ingestion of 20 g of casein, among 24 healthy older males [49].

A preliminary study on the effect of resistance exercise (RE), in combination with ingestion of a WP source (versus a control placebo group with RE alone) on myostatin and cell cycle related gene expression, demonstrated a positive effect of WP intake [50]. Another preliminary study concluded that a single session of neuromuscular electrical stimulation, prior to 20 g casein administration, was not effective in augmenting post-prandial MPS in older adults [51].

Nine RCTs further tested the effects of combination of different protein sources and RE in the elderly. The main findings of these studies are reported in Table 2 [52,53,54,55,56,57,58,59,60]. The overall results of these studies are concordant in providing evidence of the beneficial effects of RE and training, with some evidence encouraging a nutritional support with WP.

Four RCTs tested the effects of a daily administration of different protein supplements among older people along different periods (from 15 days to 2 years) [61,62,63,64]. In the USA, 15-day consecutive administration of 0.4 g/kg of body weight of tryptophan-fortified collagen to 9 older women resulted in a better nitrogen balance and lean mass than the same dose of WP [61]. In Norway, a 12-week supplementation with a protein-enriched milk twice a day (for a total of 40 g protein/day), did not improved muscle mass and strength among 50 men and women aged over 70 years [62]. In China, a 6-month trial among 180 postmenopausal women revealed a mild favorable effect on body composition of 15 g soy protein daily supplementation, in comparison to 15 g WP + 100 mg isoflavones or 15 g milk proteins [63]. In Australia, a long term trial among well-nourished 229 postmenopausal women (196 included in the analysis) did not confirm the efficacy of adding 30 g of WP in improving muscle mass and function [64].

### 3.2. Muscle Related Endpoints among Patients (14 Studies)

In this section, we considered only studies recruiting older people, addressed as patients, in relation to a condition of sarcopenia, pre-frailty or frailty or hospitalization.

A basic science study demonstrated that a leucine-enriched WP supplementation (21 g) was able to stimulate post-prandial MPS in both sarcopenic and healthy older men [65]. Further studies among sarcopenic patients investigated the effect of different nutritional supplements, in association or not with physical activity. The main findings of these studies are the following:

- during a six-month resistance training (RT) intervention among 80 mobility-limited older adults, 40 g of daily WP supplementation did not add benefit to exercise in improving lean mass, muscle strength and physical function [66];
- a leucine-enriched WP supplement with vitamin D (20 g + 800 IU, twice a day for 13 weeks) was tested versus an iso-caloric dietary supplement, and was superior to placebo in improving muscle mass and lower-extremity function among a large cohort of 380 sarcopenic older adults, even in patients who were unable to exercise [67];
- the association of physical activity with a daily supplementation consisting of WP (22 g), essential amino acids (10.9 g including 4 g of leucine) and vitamin D (100 IU) was more effective than physical activity plus placebo in increasing fat free mass and muscle strength, in improving quality of life and in decreasing inflammation index in 130 sarcopenic elderly people [68];
- the combination of regular resistance muscle training with a nutrition therapy based on an oral supplement offered twice daily (containing 20 g WP, 9 g carbohydrates, 3 g fat, 800 IU vitamin D, and a mixture of vitamins, minerals, and fibers per serving) was superior to exercise alone in improving muscle mass and strength, in 34 elderly patients at high risk of sarcopenia [69];
- the combination of RE with different isocaloric shakes containing 12 g of milk protein or 12 g of soy proteins versus placebo (rice milk, considered as non-protein control) had a positive effect on muscle mass, independently from the type of protein source (milk or soy), among 26 sarcopenic men. The same intervention study among 26 overweight sarcopenic men resulted in a decrease in fat mass only in the dairy supplemented group [70,71].

A double blind, randomized, multicenter study is taking place in Denmark to evaluate the efficacy of a ready-to-drink milk based, protein enriched supplement (27.5 g WP per day for 12 weeks), versus an iso-caloric placebo in acutely ill geriatric patients, at high risk of developing sarcopenia, recruiting 165 older adults [72]. The primary endpoint of this trial is lower extremity muscle strength and function. Up to date, no results of this trial have been published.

Two RCT assessed the effect of cow-milk protein supplements among frail patients in association or not with RT [73,74]. In the study by Dirks and colleagues, the supplementation of 15 g milk protein twice a day for 24 weeks, in association with RT, was superior to RT + placebo in augmenting muscle fiber [73]. In the study by Niccoli and colleagues, 47 hospitalized geriatric patients not able to participate in high intensity RT were randomly assigned to receive, or not, 24 g WP daily supplement, with a significant positive correlation between WP supplementation and both nutritional and rehabilitation outcome measures [74]. Similarly, in a 12-week intervention RCT among 120 frail and pre-frail old subjects at risk of malnutrition for a low baseline protein intake, participants received an individual adjusted amount of WP supplementation, to fulfil a total daily protein intake of 1.5 g/kg or 1.2 g/kg or 0.8 g/kg [75]. This study demonstrated that the total protein intake of 1.5 g/kg/day was statistically superior in improving muscle mass and physical performance without adverse effects, and suggested this protein intake goal as the most beneficial in the geriatric population.

In 2017, a Brazilian series of multicenter RCTs in pre-frail and frail elderly was launched and the rationale and protocol were published [76]. The main objective of these trials, called “Pro-Elderly Study”, is to assess the effect of 16-week intervention of different protein source supplementation, with or without RT, on the following end-points: muscle mass, strength and function, nutritional status, body composition, renal function and quality of life. The study design for pro-elderly was preceded by an exploratory trial, testing different dose combinations of WP, in association, or not, with creatine, together with RT. The preliminary trial did not support the addition of creatine in improving muscle function [77]. To date, no results for pro-elderly have been published.

In a Finnish RCT, among a cohort of 106 nursing home residents, mainly at risk of malnutrition, 20 g daily supplementation of WP vs. placebo was positively associated with maintenance of skeletal muscle mass, reduction of required assistance and improvement in general well-being [78].

### 3.3. Bones (12 Studies)

It is well known that bones deteriorate in composition, structure and function as a physiological effect of the ageing process, in both men and women [79]. The role of protein intake in this decline process is still under investigation. In our literature search, the majority of the studies addressing this health-related issue were gender specific. Although high-protein diets induce an increase in loss of calcium with urine, a pilot study among women, with a subgroup over 65 years of age, demonstrated that the calcium present in the urine is not of bone origin, and that high protein diets, up to 2.1 g/kg, are not detrimental for bone metabolism, at least in the short term [80]. Two cross-sectional large studies among 746 postmenopausal women (mean age 65 years) and 1016 older men (mean age 84 years), in Europe and USA respectively, were concordant in finding a positive association between the higher intake of animal-based and dairy protein (analyzed as a subgroup of animal-based sources) and outcomes of bone health (strength, microstructure and failure load). The studies did not find a significant association between plant-based proteins and outcomes of bone health [81,82].

Six RCTs investigated the effect of daily supplementation of different combination of protein sources, calcium, and fructans on several bone related endpoints [83,84,85,86,87,88]. The methodology and results of these trials are reported in Table 3. Despite heterogeneity in study design, milk-derived supplementation had some beneficial effects (increased insulin-like growth factor-1 (IGF-1) or reduced bone resorption markers) in 4 out of 6 RCTs.

Three RCTs among menopausal and postmenopausal women further investigated the effect of different isolated bioactive components of milk, such as lactoferrin, ribonuclease, milk basic protein fractions and caseinphosphopeptides [89,90,91]. With the exception of the caseinphosphopeptides (with neither positive nor negative demonstrated effect) [91], the supplementation of the other bioactive peptides had positive effects on osteoblastic bone formation and restoration of bone turnover, among 38 and 32 women, respectively, and for a relatively short period (6 months) [89,90].

### 3.4. Cardiovascular Diseases (Eight Studies)

Among elderly subjects, cardiovascular diseases are the worldwide leading cause of death [92]. Heart related morbidity and mortality are associated with the progressive decrease in endothelial function with ageing, possibly due to different serum level changes (in hormones, lipids, and inflammatory markers).

Three RCTs investigated the effect on vascular function of soy proteins supplementation versus milk proteins supplementation, regarded as control arm [93,94,95]. These studies tested different doses of soy protein supplementation (40, 28 and 40 g, respectively), versus different milk supplementations (casein, total milk protein and caseinate, respectively), for different study durations (3 months, 12 months and 4 weeks, respectively), and among different sample sizes of post-menopausal women (105, 202 and 18, respectively). Two studies out of three were concordant in not supporting a beneficial effect of soy on endothelial function, when compared to casein or milk protein control [93,94]. The third trial was conducted in association with a low fat/low cholesterol diet and suggested an improvement in endothelial function of isolated soy protein, compared to a caseinate supplement [95].

Two studies tested the effect of lactotripeptides, with and without regular aerobic exercise, on vascular endpoints, such as endothelium dependent dilatation and arterial compliance, among post-menopausal women [96,97]. These trials supported a beneficial effect of lactotripeptides when combined with regular physical activity.

One study tested the effect of a single dose of WP isolate on cardiovascular risk factor markers (total cholesterol, low density lipoproteins, Apolipoprotein B48, insulin levels) among 20 post-menopausal women, suggesting a possible decrease in arterial exposure to some lipoproteins [98].

A RCT tested the short-term effect of different protein-enriched supplementations among overweight postmenopausal women. Despite previous data on WP long-term supplementation showing a beneficial cardiovascular effect, the acute administration was not superior to casein or glucose on blood pressure, vascular function and inflammatory markers [99].

A long-term trial, investigating the effect on blood pressure of a two-year daily supplementation of a WP-based beverage (250 mL with 30 g proteins and 600 mg calcium) among women over 70 years, did not provide evidence of the hypotensive property of dairy proteins [100], as previously suggested in short term RCTs.

### 3.5. Protein Intake and Metabolism (Seven Studies)

A key issue in elderly nutrition is the role of dairy proteins and products as important contributors of nutrients. In 2002 and 2003, two studies assessed protein digestion and metabolism among young and old subjects. According to their results, fast proteins, WP in particular, may be beneficial in the elderly to limit protein losses during aging [101,102]. In a RCT comparing casein vs. WP supplementations among 31 elderly men, the authors outlined how postprandial protein retention was better improved by fast-digested WP [19]. In more recent years, an Australian research group explored the effect of WP supplementation in older people with malnutrition, in order to assess its effect on different endpoints: suppression of energy intake [103], gastric emptying and gut hormones response [104,105,106]. The main conclusions of these investigations were that WP supplementation in older adults increased overall protein intake without suppressing appetite, and positively affected gastric emptying and gut hormones response.

### 3.6. Inflammation Markers (Seven Studies)

The term “Inflamm-aging” was firstly used by the Italian researcher Claudio Franceschi in 2000, and it refers to the progressive and chronic development of a pro-inflammatory state with aging [107]. Increasing evidence indicates that inflamm-aging is associated with the risk of developing other diseases, such as Alzheimer’s disease, atherosclerosis, heart disease, type II diabetes, and cancer. According to this new area of research, serum markers of inflammation and the antioxidant capacity of plasma should be monitored, in order to act on the aging process.

A study tested the antioxidant capacity of a milk-based protein matrix, previously in vitro and then ex vivo, among healthy women aged 50–70 years, providing encouraging preliminary data [108].

Four RCTs evaluated the role of WP supplementation on inflammatory markers in the elderly [109,110,111,112]. Laviolette and colleagues explored the effect of 16-week daily WP supplementation vs. casein, in association with 8-week RT, among 22 patients with stable chronic obstructive pulmonary disease (COPD). They did not reported a positive effect on serum C reactive protein (CRP) or Interleukin-6 (IL-6) [109]. Among similar COPD patients, Sugawara and colleagues reported a decrease in CRP, Interleukin-8 (IL-8) and tumor necrosis factor-α (TNF α) supplemented with WP in association with exercise [110]. One trial among 31 elderly patients after acute ischemic stroke demonstrated a decrease in systemic inflammation markers with enteral formula, containing WP vs. a casein containing formula [111]. A further study among 40 community dwelling older adults, the supplementation of WP or bovine colostrum, in association with RT, did not change IGF-1 or CRP level [112].

Among 84 postmenopausal women, a multicenter 18-month supplementation trial reported a protective effect of WP on markers of inflammation, in comparison to a maltodextrin supplementation [113]. A multi-ingredient nutritional supplement was effective in progressively reducing the plasma level of TNF- α and IL-6 among healthy older men, in a two phase study with 6 weeks of supplementation alone, followed by 12 weeks of supplementation with physical exercise [114]. The multi ingredient supplement provided contained 30 g WP, 2.5 g creatine, 400 mg calcium, 500 IU vitamin D, and 1500 mg n-3 PUFA.

### 3.7. Chronic Obstructive Pulmonary Disease (Four Studies)

Within a few years, COPD is expected to become the 5th and 3rd leading cause of disability and mortality worldwide, respectively [115]. Older people with COPD, a syndrome consisting of chronic bronchitis, bronchiectasis, emphysema, and other reversible airway diseases, are at higher risk of developing complications and adverse outcomes [116].

Among eight elderly normal-weight patients with COPD, milk protein sip feeding (a drink with 8.1 g protein) was beneficial in stimulating an enhanced anabolic response, mediated by a reduction in multiple amino acids splanchnic extraction [117]. At present, available data are not conclusive about the superiority of bolus versus sip feeding of hydrolyzed milk protein mixture in stimulating the best anabolic response in older adults with COPD [118].

In a RCT among 59 elderly patients with COPD, the supplementation of 12 g of WP twice a day versus the same dose of casein reduced shortness of breath [119].

A more recent RCT with a cross-over design examined, among 23 old COPD patients, whether a free essential amino acids mixture with high level of leucine was superior in stimulating net protein gain than a similar mixture of balanced free essential and non-essential amino acid naturally present in WP [120].

### 3.8. Neurocognitive Function (Four Studies)

Age-related cognitive decline (ARCD) is one of the main increasing concerns in an ageing population, affecting to varying degrees about 40% of people above 60 years, even in physically healthy conditions [121]. Two RCTs evaluated the effect of milk protein and WP supplementation on neuro-cognitive endpoints in elderly subjects [122,123]. In the first RCT, 15 g of a milk protein concentrate supplementation was superior to placebo in improving reaction time among 65 old people, without improving other cognitive functions [122]. In the second study, 50 g of WP isolate (WPI) was tested, versus the same amount of soy protein isolate (SPI), in a cross-over intervention among 56 elderly subjects. The study revealed an improvement of vitamin B12 and folate status, without a direct effect on cognitive status in WPI phase, and a better reaction time and reasoning speed among female subjects (but not among males) during SPI phase [123].

In a randomized, placebo-controlled, double-blind clinical trial involving 15 healthy middle-aged and older adults, the supplementation for eight weeks of lactotripeptide, a milk protein-derived bioactive peptide, increased middle cerebral blood flow velocity, an outcome associated with lower cerebrovascular disease [124].

A still ongoing RCT, the Phospholipid Intervention for Cognitive Ageing Reversal (PLICAR) study, is investigating the effect of a phospholipid-rich milk protein concentrate among old people with an age-related memory impairment [125]. To date, no results of this trial have been published.

### 3.9. Response to Vaccines (Two Studies)

Immunosenescence is defined as a progressive and physiological decline of the immune system function associated with aging, and it may start as early as the age of 55 years [126]. The impaired functioning of both innate and acquired arms of the immune system leads to the increased incidence of infections as well as higher rates of complications and fatal events among elderly population [127]. The immunosenescence is furthermore responsible for a decreased immunogenicity following vaccination. For this reason, improving vaccine efficacy in the elderly has become a global public health issue [128]. Several investigations have been performed to find environmental factors, such as immune-stimulatory food in diet, which can help the ageing immune system to drive an appropriate response to infections and vaccination [129,130,131,132,133].

Two randomized, multicenter, double-blind, controlled clinical trials were performed among 86 and 222 elderly volunteers over 70 years of age, in a pilot and confirmatory study, respectively. The aim of these trials was to assess the effect on the immune response to influenza vaccination of a daily supplementation of a probiotic dairy drink for 7 and 13 weeks, respectively. Both trials demonstrated an increased relevant specific antibody response in the probiotic group in comparison with the control group, treated with a non-fermented dairy product [134].

The antibody immune response to *Streptococcus pneumoniae* (or pneumococcus) vaccine is a good marker of the immune function [135]. Based on this assumption, a randomized, controlled, double blind pilot study was conducted among 17 healthy subjects, aged over 60 years, to assess the efficacy in improving immune response to vaccine of an 8-week daily supplementation with 5 g of WP, versus 5 g of soy proteins in the control group [136]. After the administration of pneumococcal vaccine, subjects receiving WP supplementation had a higher serum response against 12 out of 14 pneumococcal types, especially for the four most virulent bacterial types.

A recent randomized, controlled, double blind pilot study was performed among 21 healthy subjects over 60 years of age. It compared 12 g of daily consumption of milk proteins and 12 g of soy proteins for an 8-week period, focusing on the response to Diphtheria, Tetanus, and Pertussis (DTaP) vaccine as the primary endpoint [137]. The study revealed a significant increase in tetanus antibodies in the dairy group compared to the soy group.

### 3.10. Miscellanea (5 Studies)

Preliminary reports have been published in other investigational areas, and the main findings are reported in Table 4 [138,139,140,141,142].

## 4. Discussion

To our knowledge, this is the first systematic literature review targeting the role of milk protein supplementation in the elderly. The large majority of the studies included in our review showed a beneficial effect of milk protein supplementation, whey proteins in particular, in promoting improved health outcomes in a wide range of body systems. The variety of outcomes reflects the multidimensionality of diet-based support therapies. This review clearly reveals the lack of long-term studies and the need for further research on the persistency over time of the beneficial effects of milk proteins supplementation, as well as on the late onset of possible side effects.

As stated in the guidelines on clinical nutrition and hydration in geriatrics by the European Society of Clinical Nutrition and Metabolism (ESPEN), nutrition is a key factor in preserving health and wellbeing in old people [143]. Dairy products provide not only proteins, but also a substantial amount of micronutrients (vitamins and minerals) relevant for healthy ageing, and according to some authors, reference national food patterns should consider their unique nutritional properties, especially for frail elderly people [144]. Several studies included in this review were concordant in identifying WP as good nutritional sources in ageing population, especially because they are fast digested and well absorbed, rich in essential amino-acid and in leucine, and able to stimulate muscular protein synthesis without suppressing overall energy intake or increasing fat mass. These characteristics are very relevant to older people, taking into account that muscle loss (in mass and in strength), anorexia and sarcopenia, with and without obesity, are frequent among this population group.

The main finding of this review is the evidence of the role of milk protein supplementation in the maintenance of skeletal muscle mass in ageing. Studies carried on up to 24 weeks showed that milk protein supplementation increased serum level of amino acids and leucine during the post-prandial period, resulting in leucine uptake by muscle cells, myofibrillary protein and mitochondria protein synthesis in both resting and active muscles. The increased MPS was demonstrated in the elderly, with and without adequate dietary protein intake. Most studies demonstrated the superiority of WP (fast proteins) to casein (slow proteins), soy or wheat proteins. It should be noted that the only long-period study (two years) included in this review, about this research area, did not show impact on muscle mass of WP supplementation in well-fed postmenopausal women [64]. Further long-term studies are mandatory, because there are still concerns about excessive protein load and related negative effects on kidney and ageing process [145].

Strictly linked to muscle loss, the role of milk protein in sarcopenia, “one of the most meaningful geriatric syndromes” [146], was addressed by several studies included in this review. There is consensus on three fundamental factors in the prevention of sarcopenia: exercise (‘*use it or lose it!*’), quantity of protein in the diet (20–30 g of proteins per main meal) and quality of proteins (preferring high leucine content, 4 g per meal) [147]. The ideal moment of protein intake could be after exercise, since physical exercise boosts blood circulation, which increases the absorption of leucine. Protein supplementation in patients with sarcopenia is strongly recommended in the absence of other medical contraindications, especially when reaching protein needs through diet modifications alone are unsuccessful [6]. There is an urgent need of evidence-based strategies aimed to improve recovery after hospital discharge in older adults. Exercise, probably the best way to recover muscle mass, is not always applicable, so efforts towards finding efficient nutritional strategies are expected. Preliminary findings [74] reported that 24 g of WP supplementation during a daily rehabilitation program have an impact in promoting better health outcomes. Future studies designed to incorporate longer-term intervention, or post-hospitalization lifestyle modifications, are warranted.

Most studies included in this review focused on the effects of milk protein supplementation on bone metabolism are gender specific, enrolling mainly postmenopausal women. In intervention studies, milk proteins have proven to be superior to soy proteins in reducing bone resorption. Promising results indicate that the increase in serum IGF-1 levels induced by milk protein supplements was accelerated, with a significant difference, by zinc addition [148].

The only long-term trial investigating the effect on blood pressure of two-year daily supplementation of a WP-based beverage among older women did not provide evidence of a hypotensive property of dairy proteins [100], as suggested by short term RCTs.

The protective role of milk protein supplementation on serum markers of inflammation is still under investigation: the RCTs included in this review are not concordant. According to a study by Ticinesi and colleagues, further large studies assessing the anti-inflammatory effect of combined dietary supplements, including n-3 PUFA, vitamin D and WP, are needed [149].

Milk protein supplementation for old COPD patients may have multiple positive therapeutic outcomes, thanks to their role in enhancing anabolic response [117] and attenuating perceived exertion during exercise [110]. Better results may be obtained, combining nutritional intervention with supervised exercise training, as part of a formal pulmonary rehabilitation program [150].

Results of milk protein supplementation to prevent or ameliorate ARCD and dementia are still contradictory. Limited evidence showed some improvements in reaction time [122], but no significant effects have been recorded on other cognitive functions. These results are probably due to the wide spectrum of neurocognitive manifestations with different underlying physiopathology mechanisms, which makes it difficult to standardize intervention protocols. As stated in a systematic review published in 2011, several research projects were involved in finding effective dietary interventions, which aimed to prevent or ameliorate ARCD [151]. The main conclusion of the review is that low-fat dairy products, together with an adequate diet, may play a beneficial role in neurocognitive function during aging.

Milk protein supplementation is a promising nutritional intervention to stimulate immune response [136,137]. Further studies are advocated, to investigate its role in counteracting immune-senescence, in reducing inflamm-aging and in stimulating response to infections and vaccination. The role of fermented dairy products, with and without milk protein supplementation, could represent an interesting starting point for further investigation, given the high compliance reported in this type of intervention [132,134].

One of the major strengths of this review is the wide overview on several health topics related to ageing. A narrative synthesis of 103 articles gave the opportunity to (i) discuss the evidence on key research areas as well as on poorly investigated ones, (ii) map different target population, mainly healthy subjects, but also frail, sarcopenic, hospitalized and chronically ill patients, (iii) include gender medicine studies, and (iv) underline some knowledge gap.

Other key strengths of our work are the robust methodology adopted according to PRISMA criteria, the approved registration on PROSPERO, the extensive research through five electronic databases, including Clinicaltrials.gov to overcome publication bias, and a further search in Google Scholar. Moreover, two independent investigators (BZ and MZ) screened titles and abstracts to select included studies.

We acknowledge some limitations. We did not provide any meta-analysis, the heterogeneity of the included studies was explored descriptively. We did not conduct an a priori quality assessment of the studies, because we preferred including all studies selected according to the pre-defined objective criteria, without introducing any subjective judgements.

## 5. Conclusions

Our systematic literature review supports the evidence of a beneficial effect of cow-milk protein supplementation among old people. Evidence of beneficial effects is stronger for whey proteins supplementation. Several health outcomes are reported with respect to milk proteins: achievement of nutritional aged-related goals, muscle mass preservation and functionality, prevention and treatment of sarcopenia, modulation of inflammation, response to vaccinations and rehabilitation after hospitalization.

## Figures and Tables

**Table 1:** Studies aimed to identify the best protein supplementation in inducing muscle protein synthesis or accretion, after acute administration.

Author, Year	Number Participants, Gender	Age (Mean or Range)	Type of Study: Intervention Arms	Main Endpoints	Results
Paddon-Jones, 2006 [30]	14, 7 ♀/7 ♂	68	RCT: 15 g WP vs. 15 g EAA	Muscle FSR for 3.5 h after ingestion	Both supplementations stimulated FSR, with greater increase in EAA arm
Katsanos, 2008 [31]	15, 6 ♀/9 ♂	60–85	RCT: 15 g WP vs. 6.72 g WP’s EAA vs. 7.57 WP’s Non-EAA	blood phenylalanine, insulin, glucose concentration, muscle biopsy	WP improves MP accrual through mechanisms beyond its EAA content
Koopman, 2009 [32]	10, ♂ (cross over)	64	Case-control study: 35 g intact casein vs. 35 g hydrolyzed casein	blood phenylalanine concentration, muscle biopsy (FSR)	Hydrolysate accelerates protein digestion and absorption, increase AA availability and FSR
Pennings, 2011 [33]	48, ♂	74	RCT: 20 g WP vs. 20 g casein vs. 20 g casein hydrolysate	Postprandial Muscle FSR	MP accretion more effective in WP arm
Burd, 2012 [34]	14, ♂	71	RCT: 20 g micellar casein vs. 20 g WP	Rate of MPS at rest and after exercise	Greater rates of MPS in WP arm
Groen, 2012 [35]	16, ♂	74	RCT: intra-gastric administration during sleep of 400 mL of water with vs. without 40 g casein	BPB, MPS	Casein administration during sleep improves BPB and stimulates MPS
Pennings, 2012 [36]	33, ♂	73	RCT: 10 g vs. 20 g vs. 35 g WP	AA absorption, BPB, MPA	35 g WP reaches best values in all endpoints
Wall, 2013 [37]	24, ♂	74	RCT: 20 g casein vs. 20 g casein + 2.5 g leucine	MPA	Leucine co-ingestion improves MPA
Luiking, 2014 [38]	19, 10 ♀/9 ♂	69	RCT: 20 g WP vs. 6 g milk protein, both arms after unilateral resistance exercise	MPS	Higher MPS with WP, without further enhance with exercise
Churchward-Venne, 2015 [39]	32, ♂	71	Parallel group study: 25 g casein in milk matrix vs. 25 g casein in water	Post-prandial MPS	Milk matrix delays casein digestion and absorption without affecting MPS
Borack, 2016 [40]	20, ♂	55–75	RCT: 30 g WP isolate vs. 30 g soy-dairy protein blend (25% soy, 25% WP and 50% casein); both arms after resistance exercise	Blood and muscle AA concentration; FSR	No differences in endpoints among arms
Gorissen, 2016 [41]	60, ♂	71	RCT: 35 g WhP vs. 35 g WhPH, vs. 35 g micellar casein vs. 35 g WP vs. 35 g WPH vs. 60 g WhP	Post-prandial AA concentration and MPS	Greater AA concentration after WP, greater MPS after micellar casein
Walrand, 2016 [42]	31, ♂	72	RCT: 10-day period of AP or HP diet followed by ingestion of 15 g or 30 g casein vs. 15 g or 30 g of soluble milk proteins	FSR	Greater increase in FSR after ingestion of soluble milk proteins only in the AP group
Kouw, 2017 [43]	48, ♂	72	RCT: before sleep administration of 40 g casein vs. 20 g casein vs. 20 g casein + 1.5 g leucine vs. placebo	MPS	Ingestion of 40 g casein increases MPS better than other arms
Hamarsland, 2019 [44]	21, 8 ♀/13 ♂	74	RCT: 20 g WP vs. 20 g native WP vs. milk (ingested after 2 h of resistance training)	Serum leucine concentration; FSR	Greater increase in serum leucine in native WP arm, but no difference with WP in FSR (only superior to milk)

♀: females; ♂: males; RCT: Randomized Clinical Trial; WP: Whey Proteins; EAA; Essential Amino Acids; FSR: Fractional Synthetic Rate; MP: Muscle Protein; MPS: Muscle Protein Synthesis; BPB: Body Protein Balance, AA: Amino Acids; MPA: Muscle Protein Accretion; WhP: Wheat Protein; WhPH: Wheat Protein Hydrolysate; WPH: Whey Protein Hydrolysate; Adequate Protein; HP: High Protein.

**Table 2:** Randomized Clinical Trials testing the effects of different protein supplementation in combination with exercise training.

Author, Year	Number Participants, Gender	Age (Mean or Range)	Duration	Intervention Arms	Main Endpoints	Results
Dideriksen 2011 [52]	24, 9 ♀/15 ♂	68	acute supplementation	RE + WP (0.45 g/kg) vs. RE + caseinate (0.45 g/kg)	MPS	Increase in MPS, no difference between arms
Yang 2012 [53]	37 ♂	71	acute supplementation	WP 0 g, 10 g, 20 g or 40 g vs. WP same doses + RE	MPS	RE increases MPS at all WP doses with greater extent with 40 g WP
Arnarson 2013 [54]	161, 94 ♀/67 ♂	65–91	12 weeks	RE + WP (20 g) vs. RE + isocaloric CHO	Lean body mass, strength, physical function	Increase in all endpoints, no difference between arms
Gryson 2014 [55]	48, ♂	61	16 weeks (sedentary)	MET + total milk proteins (10 g) vs. MET + soluble milk proteins rich in leucine (10 g)	Muscle mass and strength, time to task failure, index of muscle fatigue	Better results in all endpoints with soluble milk proteins + after MET
Karelis 2015 [56]	99, 76 ♀/23 ♂	65–88	135 days	20 g of cysteine enrich-WP vs. 20 g casein (both arms in combination with RT)	Body composition (DXA), muscle strength	Muscle strength increases in both arms, additional increasing WP arm
Weisgarber 2015 [57]	12, ♀	57	10 weeks	RE (high volume) + WP (40 g) vs. RE + placebo	Lean tissue mass, muscle thickness, muscle strength	Increase in muscle thickness and strength, but no difference between arms
Thomson 2016 [58]	179, 99 ♀/80 ♂	62	12 weeks	RE + high dairy protein (1.2 g/kg) vs. RE +high soy protein (1.2 g/kg) vs. RE + usual protein intake (<1.2 g/kg)	Muscle strength, body composition, physical function, quality of life	Increase in lean mass, physical function and mental health in all arms, increase in strength attenuated in soy arm
Mori 2018 [59]	81, ♀	65–80	24 weeks	RE + WP (22.3 g/day) vs. RE alone vs. WP alone	Muscle mass, physical function	Higher improvement in all endpoints in RE + WP arm
Sugihara 2018 [60]	31, ♀	67	12 weeks	RE + WP (35 g) vs. RE + placebo	Muscle strength, hypertrophy, muscle quality	Higher increase in muscle strength and hypertrophy in RE + WP

♀: females; ♂: males; RE: Resistance Exercise; WP: Whey Protein; MPS: Muscle protein Synthesis; CHO: Carbohydrates; MET: multicomponent exercise training; RT: Resistance Training; DXA: Dual energy X-ray Absorptiometry.

**Table 3:** RCTs investigating the effect of daily supplementation of different combination of protein sources, calcium and fructans on several bone related endpoints.

Author, Year	Number Participants, Gender	Age Mean and/or Range	Duration	Intervention Arms	Main Endpoints	Results
Khalil 2002 [83]	17, ♂	65–84	3 months	Daily supplementation of 40 g SP vs. 40 g MP	Bone specific ALP activity, urinary deoxypyridinoline excretion	No endpoint difference among arms
Holm 2008 [84]	29, ♀ Postmenopausal	55	24 weeks	10 g WP + 31 g CHO + 1 g fat + 5 mcg vitamin D + 250 mg calcium vs. 6 g CHO + 12 mg calcium; both arms with ST	BMD with DXA, Osteocalcin, CTx	Increase in BMD and osteocalcin in WP multi-ingredient arm
Adolphi 2009 [85]	85, ♀ postmenopausal	59 (48–67)	2 weeks	Bedtime consumption of 175 mL Fm vs. 175 mL Fm + 510 mg Calcium vs. Fm+ 510 mg calcium + 0.175 g CPP + 1.75 g ITF	Nocturnal bone resorption markers	Fm reduced bone resorption independently of further supplementation
Chevallley 2010 [86]	45, ♀ (recent hip fracture)	81	1 week	Daily supplementation of 20 g casein vs. 15 g WP vs. 5 g EAA	Elevation of circulating IGF-1	Increase in IGF-1 in casein arm supplementation
Zhu 2011 [87]	219, ♀	70–80	2 years	Daily supplementation of a drink with 30 g WP vs. placebo	BMD with DXA and QCT. IGF-1 level, urinary calcium excretion	Increase in IGF-1 at year 1 and 2 in WP arm, but no effect on bone mass or strength
Kerstetter 2015 [88]	208, 178 ♀/30 ♂	70	18 months	Supplementation of 45 g WP vs. isocaloric placebo	BMD with DXA, fat free mass	No difference in BMD, better preservation of fat free mass in WP arm

♂: males; ♀: females; SP: Soy Protein; MP: Milk Protein; ALP: Alkaline Phosphatase; WP: Whey Protein; CHO: Carbohydrates; ST: Strength Training; BMD: Bone Mineral Density; DXA: Dual energy X-ray Absorptiometry; CTx: C-Terminal telopeptide, Fm: Fermented milk; CPP: Casein Phospho-peptides; ITF: Inulin-Type Fructans; EAA; Essential Amino Acids; IGF-1: Insulin-like Growth Factor-1; QCT: Quantitative Computed Tomography.

**Table 4:** Preliminary reports have been published in other investigational areas.

Author, Year	Study Type	Intervention	Main Endpoints	Results	Brief Comment
Numan 2007 [138]	Pilot study	Anti-Clostridium difficile WP concentrate	Prevention of relapse of Clostridium difficile infection	10% relapse rate in comparison to 20–25% relapse rate in a control contemporary cohort	Waiting for confirmation in RCT
Coker 2012 [139]	RCT during caloric restriction	Meal replacement with WP and EAA vs. a standard meal replacement	Weight loss preserving lean tissue (muscle mass)	WP + EAA was effective in weight reduction promoting preferential reduction of adipose tissue	Small sample size (12 subjects)
Ooi 2015 [140]	RCT	30 g WP supplementation vs. a high CHO energy match supplementation	Weight reduction and reduction of hepatic steatosis in women	No difference in weight reduction or hepatic steatosis	WP supplementation may reduce hepatic steatosis despite weight gain
Dhillon 2017 [141]	RCT crossover design	WP isolate (50 g) vs. soy protein isolate (50 g)	Bioavailability of folates and Vitamin B12 in elderly with subclinical deficiencies	WP isolate was superior to soy in improving active B12 and folate status	-
Song 2018 [142]	Blind sensory analysis	Rye bread and cream cheese enriched with: - WP hydrolysate - WP isolate - Soy protein isolate	Consumer acceptance	Better acceptance of WP hydrolysate in bread and of WP isolate in cheese	Developing protein enriched food may increase protein intake in elderly but innovation in protein enriched appealing food is challenging

RCT: Randomized Control Trial; WP: Whey protein; EAA: Essential Amino Acids; CHO: Carbohydrates.
